# Progress on the Synthesis Pathways and Pharmacological Effects of Naturally Occurring Pyrazines

**DOI:** 10.3390/molecules29153597

**Published:** 2024-07-30

**Authors:** Xun Liu, Wenli Quan

**Affiliations:** College of Bioengineering, Sichuan University of Science & Engineering, Yibin 644000, China; xunliu0123@hotmail.com

**Keywords:** pyrazine, 2,3,5,6-tetramethylpyrazine, 2,5-dimethylpyrazine, 2,3,5-trimethylpyrazine, biosynthesis, pharmacological effect

## Abstract

As one of the most essential types of heterocyclic compounds, pyrazines have a characteristic smell and taste and have a wide range of commercial applications, especially in the food industry. With the development of the food industry, the demand for pyrazines has increased. Therefore, understanding the properties, functions, and synthetic pathways of pyrazines is one of the fundamental methods to produce, control, and apply pyrazines in food or medical systems. In this review, we provide an overview of the synthesis pathways and physiological or pharmacological functions of naturally occurring pyrazines. In particular, we focus on the biosynthesis and pharmacological effects of 2,3,5,6-Tetramethylpyrazine (TTMP), 2,5-Dimethylpyrazine (2,5-DMP), and 2,3,5-trimethylpyrazine (TMP). Furthermore, areas where further research on pyrazines is needed are discussed in this work.

## 1. Introduction

Pyrazines, as a class of volatile compounds containing heterocyclic nitrogen, have been identified as crucial flavoring constituents in traditional fermented foods, such as the Chinese liquor *Baijiu*, cheese, and soy sauce, which contribute to roasted, baked, and nutty flavors [1,2]. Natural pyrazines are alkaline with monocyclic aromatic rings, which can be substituted with alkyl, acyl, methoxyl, sulfur-containing thiol, or sulfide groups at different positions [3,4]. These different substitution types contribute to the formation of different flavors [4]. Among them, alkylpyrazines have become important flavoring agents in the food industry, with non-mutagenic and non-carcinogenic characteristics [5]. Ma et al. [6] investigated the interaction between bovine serum albumin (BSA) and pyrazine homologues, including 2-methylpyrazine, 2,5-DMP, TMP, and TTMP. The results elucidated that the distribution of alkyl in a pyrazine ring influences its flavor release in a BSA solution by inducing conformation and polarity change in BSA, which in fact affect the aroma release of pyrazines in food materials [6].

As important heterocyclic flavor compounds, pyrazines are mainly formed from fermentation processes or the Maillard reaction and present a baked or roasted flavor, which is beneficial to enhance the flavor quality of food, including in flavoring, meat production, etc. [6,7,8,9]. Zhao et al. [10] reported that the formation of pyrazines related to the Maillard reaction during food thermal processing came down to the formation of an aminoketone group derived from amino acids and α-dicarbonyl groups. Previous studies revealed that peptides served as precursors in the formation of alkylpyrazines [11]. Pyrazines can be extracted from natural sources (such as animals, insects, and plants), but this is often limited by a low concentration and impurity [4,12]. Furthermore, the high costs for extraction and the risk of taste and smell changes also affect industrial production of pyrazines [12].

In chemical synthesis pathways, L-serine, L-threonine, or a mixture of the two are used as substrates to generate pyrazines at either a low temperature (120 °C for 4 h) or high temperature (300 °C for 7 min) after going through a variety of chemical reactions, such as dehydration, deamination, decarbonylation, aldol-condensation, and Strecker degradation [13]. It has been proved that different substrates can form different types of pyrazines [13]. Specifically, 2,5-DMP, 2,6-DMP, TMP, and 2-ethyl-3,5-dimethylpyrazine are derived from L-threonine, while ethylpyrazine, methylpyrazine, and 2,6-diethylpyrazine are derived from L-serine [13].

The biosynthesis of pyrazines with microorganisms is environmentally friendly compared to chemical synthesis [14]. Two *Bacillus subtilis* strains were isolated from fermented soybeans by Kłosowski et al. [14], which were able to produce 2-methylpyrazine, 2,3-DMP, 2,6-DMP, 2,5-DMP, 2,3,5-TMP, and TTMP. In addition, their results revealed that different *B. subtilis* strains were inclined to produce different types of alkylpyrazines [14]. In previous studies, several pyrazines and acyloin derivatives were detected in the volatiles released by *Corynebacterium glutamicum* [15]. Through performing feeding experiments with labeled acetoin together with using genetic engineering technology, researchers studied the primary metabolism of pyrazine biosynthesis and constructed a preliminary model of the generation of alkylated pyrazines via acyloins [15]. By performing isotope feeding experiments, Silva-Junior et al. [16] found out that L-threonine together with sodium acetate were precursors in pyrazines’ biosynthesis pathways. Furthermore, to some extent, supplementing with high concentrations of L-threonine can induce the production of pyrazines [16].

Pyrazines are important types of aroma compounds in the Chinese liquor *Baijiu*, and they are crucial for maintaining the quality of *Baijiu* [17,18]. As six-membered heterocyclic compounds, pyrazines have two nitrogen atoms at positions 1 and 4, which are important classes of aroma compounds in *Baijiu* [18,19]. In one study, 16 pyrazines in total were examined in soy-sauce-aroma-type *Baijiu* samples, and the results showed that 2,6-DMP, TMP, and TTMP were the three most abundant pyrazines [20]. Several sub-threshold pyrazines (including 2,3-DMP, 2-acetyl-3-methylpyrazine, and 2,3-diethylpyrazine) are significantly connected with the roasted aroma in soy-sauce-aroma-type *Baijiu*, despite the fact that their concentrations are below the threshold [20]. High yields of pyrazines have been detected in three *B. cerberus* strains selected from soy-sauce-flavor *Baijiu* during the fermentation process [21]. These results showed that pyrazines cannot be detected in the first 48 h, while their production approached the peak level after 144 h in the wheat fermentation medium [21].

Pyrazines can be applied in many fields depending on their various functions. Pyrazines, especially alkylated pyrazines, have gained more and more attention from the food industry, due to their perceived odor characteristics, and have been applied in raw, roasted, or microwave foods as key ingredients [12]. Simultaneously, alkylpyrazines can also function as food preservatives due to their effective antimicrobial characteristics [4]. Due to the chemical properties of pyrazines, these types of compounds and some of their derivatives have been used to produce insecticides, pesticides, dyes, and pharmaceuticals [14]. Pyrazines have been identified as trail and alarm pheromones in ants, which were produced by leaf-cutter ant-associated bacteria strains [16]. Pyrazines found in insects (e.g., *Phyllium westwodii* and *Coccinella septempunctata*) and plants (e.g., *Pisum sativum*) serve as odor signals, contributing to scaring predators and protecting fruits and vegetative tissues of plants from being eaten, respectively [14,15,22]. A direct-immersion-based stir bar sorptive extraction methodology was established by Rigling et al. [23] and utilized to detect alkylpyrazines in several types of tea. Their results revealed that 2-methylpyrazine and 2,5-DMP were the most abundant alkylpyrazines in the tea samples and indicated that the differentiable compositional pattern of alkylpyrazines potentially contributed to distinguish different types of tea [23]. In addition, it has been reported that pyrazine and its derivatives are effective corrosion inhibitors for industrial alloys and metals [24]. Pyrazines can be used as antimicrobial agents for plants, animals, and humans [25]. Zou et al. [26] reported that TTMP and its derivatives functioned as suppressors of inflammation and bacterial growth. 2,5-DMP can decrease non-esterified fatty acid levels in the plasma of rats [27]. In addition, pyrazine derivatives are also widely used in the medical field. For example, pyrazinamide and sulfametopyrazine can be used for antimycobacterial purposes, while acipimox and oltipraz can be used for decreasing blood lipids and for their antitumor behavior, respectively [25,28].

**Figure 1:**
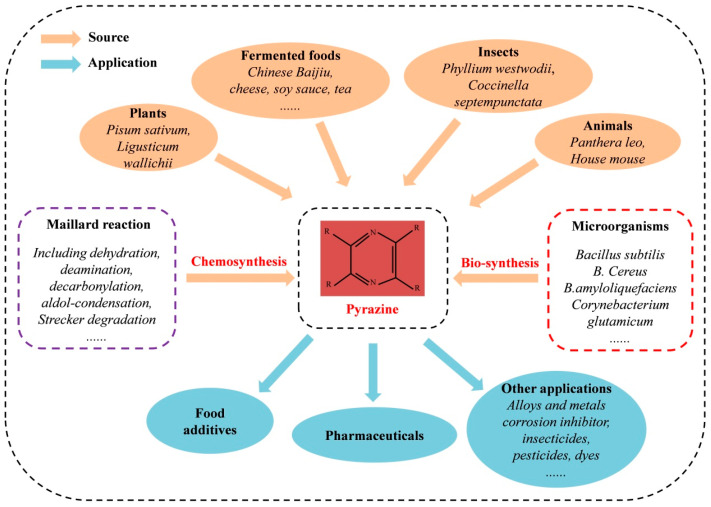
Summary of sources and applications of naturally occurring pyrazines.

The applications of pyrazines are becoming more and more wide and demand is increasing, especially in the booming food industry. The sources and applications of pyrazines are summarized in Figure 1. In this review, we focused on several kinds of naturally occurring vital pyrazines, including TTMP, 2,5-DMP, and TMP, which play crucial roles in the fast food industry and have important influences on the quality of the Chinese liquor *Baijiu*.

## 2. Naturally Occurring Pyrazines and Their Pharmacological Applications

### 2.1. Tetramethylpyrazine

2,3,5,6-Tetramethylpyrazine (TTMP), also known as ligustrazine, is a biologically active chemical containing nitrogen originally isolated from a Chinese herbal medicine *Ligusticum wallichii* [29]. TTMP has been detected in different fermented foods, such as soya sauce, vinegar, sweet wine, beer, miso, sake, and *Baijiu*, which are produced by different microorganisms [4,30,31]. TTMP works as a functional ingredient in vinegar, and the concentration of TTMP is identified as a contributing factor in the quality of vinegar [32]. As a colorless compound with a roasted-nut flavor, TTMP has been considered as a safe substance and widely used as a flavor additive in the beverage and food industries [33,34]. In previous studies, TTMP was said to likely aid in treating cardiovascular and cerebrovascular diseases [35]. Recently, researchers found that TTMP had a beneficial effect in treating several diseases, such as pulmonary hypertension, endometriosis, spinal cord injury, Alzheimer’s disease, and liver injury [34,36,37,38,39,40].

#### The Potential Medical Effects of TTMP

In the medical field, TTMP has a relatively large ability to maintain its wide range of applications in the future, since a lot of scientific studies have been carried out on this aspect (Table 1). TTMP produced by *B. amyloliquefaciens* XJB-104 had a potential role in liver protection when TTMP was placed in an ethanol–water system at a low dose [39]. In detail, TTMP significantly decreased serum levels of biochemical indicators of liver injury, including aspartate aminotransferase, alanine aminotransferase, lactate dehydrogenase, and alkaline phosphatase [39]. TTMP together with paclitaxel can inhibit ovarian tumor cell proliferation by suppressing angiogenesis and promoting cancer cell apoptosis [41]. Meanwhile, TTMP can also further enhance the antitumor effects of paclitaxel [41]. Zou et al. [42] reported that TTMP and paclitaxel delivered by a redox-sensitive nano-system had synergistic efficacy for suppressing cancer cells’ growth. In a separate study, researchers found that TTMP treatments effectively relieved the symptoms of Alzheimer’s disease through changing the expression of relevant proteins (related to oxidative phosphorylation) in the hippocampus of mice [36]. Similarly, due to the ability to increase the level of synapse-related proteins in the hippocampus and relieve dendritic and spine deficits, TTMP is capable of alleviating anxiety, improving recognitive ability, and reducing memory impairments [38]. The antioxidant, anti-inflammatory, and neuro-protective activity of TTMP can contribute to treat spinal cord injury [43]. Lin et al. [44] established a middle cerebral artery occlusion rat model to study the mechanism of TTMP in treating ischemic stroke. The results suggested that TTMP can improve neurological function through synaptic ultra-structure remodeling and by increasing synaptophysin levels [44]. Similarly, subsequent research found that TTMP can ameliorate neurological functional recovery and enhance endogenous neurogenesis and angiogenesis of rats with subacute ischemia [45]. TTMP also played a role in treating endometriosis, owing to its function of decreasing lesional fibrosis and lesion weight [40]. Due to the antioxidant properties of TTMP, inhaled TTMP aerosols prevented the progression of pulmonary hypertension in a rat model [37]. Min et al. [46] reported that TTMP has a therapeutic role in preventing acute lung injury through protecting the pulmonary microvascular endothelial cell barrier and suppressing inflammation. In addition, Yang et al. [47] summarized the inhibitory effect of TTMP on different tumors. 2-[[(1,1-dimethylethyl)oxidoimino]-methyl]-3,5,6-trimethylpyrazine (TBN) as a novel derivative of TTMP has a free-radical scavenging nitrone moiety, which contributes to treating motor deficits in mice by activating mitochondrial antioxidant activity [48]. A series of TTMP derivatives were designed and synthesized as antiplatelet aggregation agents, which were beneficial to the treatment of ischemic stroke [49]. TTMP and its derivatives had neuroprotective effects in diseases of the central nervous system through multifaceted mechanisms [50]. To sum up, the reported physicochemical properties of TTMP contribute to its potential comprehensive capabilities in the medical field.

### 2.2. Dimethylpyrazine

Dimethylpyrazines have multiple types with different substituent groups, in which the most common ones are 2,5-DMP, 2,3-DMP, 2,6-DMP, etc., and they often appear in fermented soybeans and cocoa beans, bakery and potato products, rum and whiskey, respectively [4,51,52]. It has been reported that 2,5-DMP is one of the most dominating pyrazines in fermented food [4]. Therefore, in this review, we focused on 2,5-DMP, specifically coming down to its characteristics, biosynthetic pathways, and potential medical functions.

2,5-DMP has a strong aroma of roasted peanuts, chocolate, and cream, and it has been widely discovered in heat-treated and traditional-fermented foods, including *Baijiu*, coffee, tea, nuts, roasted foods, and so on [53,54,55,56]. Furthermore, with its unique aroma characteristics, 2,5-DMP constitutes the dominating flavor component in steamed crabs (*Eriocheir sinensis*) [57]. Under normal environmental conditions, 2,5-DMP can been oxidized with a high probability [58]. Therefore, this chemical property affects its applications in the food industry to a large extent [58]. To extend its residual action and enhance the thermo-stability of 2,5-DMP, hydroxypropyl-b-cyclodextrin (HP-b-CD) has been used as a wall material to bundle 2,5-DMP, forming a spherical, flavored nanocapsule; through this process, 2,5-DMP can become a promising, high-quality food spice in the future [58]. 2,5-DMP has been detected in dog food by Chen et al. [59], who demonstrated that exogenously added 2,5-DMP can remarkably enhance the tastiness of dog foods.

#### The Potential Medical Effects of 2,5-DMP

2,5-DMP not only has important economic value in the food industry as a type of essence, but it also has potential effects in the pharmaceutical field as an intermediate [60] (Table 2). It has been reported that 2,5-DMP is capable of inhibiting the growth of the uterus of rats [61]. Furthermore, 2,5-DMP also remarkably reduced the absorption of 3H-estradiol in the uterus of rats [61]. Thereafter, the effects of DMP isomers on reproductive organs were studied in male rats as well [62]. The results showed that 2,5-DMP had a negative impact on the weight of the prostate and seminal vesicle by inhibiting testosterone uptake and decreasing plasma testosterone, while 2,6-DMP only effected the weight of seminal vesicles and 2,3-DMP had no effect on accessory reproductive organs [62]. Yamada et al. [63] found out that 2,5-DMP had a role in decreasing the level of circulating testosterone, resulting in a reduction in polyamines and acid phosphatase in the prostate in juvenile male rats, although it had no influence on mature male rats. 2,5-DMP had a role in prolonging the therapeutic sleep time induced by phenobarbital in mice, as well as in strengthening the GABAnergic system in the brain of mice [64]. A low dose of ritodrine hydrochloride together with 2,5-DMP had an inhibitory function on uterine contraction in female rats [65]. As a pheromone for female house mice, 2,5-DMP significantly increased the probability of sperm-head abnormalities in male mice [66]. Kagami et al. [67] reported that a lipid-lowering therapy by nicotinic acid induced a rebound phenomenon in plasma non-esterified fatty acid concentration. However, co-application of 2,5-DMP with nicotinic acid showed continuous low concentrations of plasma non-esterified fatty acid, which indicated that 2,5-DMP may be conducive to suppressing the non-esterified fatty acid rebound induced by nicotinic acid [67]. Similarly, 2,5-DMP, 2,3-DMP, 2,6-DMP, and their metabolites showed blood lipid-lowering effects in rats [68]. It has been reported that the application of 2,5-DMP decreases the plasma non-esterified fatty acid levels in rats [27]. Summarized above is the previous research on the potential medical effects of 2,5-DMP; however, in recent years, relevant research reports have been scarce.

In addition, 2,5-DMP can be useful in other ways. 2,5-DMP in the urine of carnivorous ferrets worked as a kind of odor signal, which was beneficial for recognition by their prey, dwarf hamsters [69]. Secretion or marking fluid, released by animals, containing 2,5-DMP has been identified as a chemical pheromone, which plays a role in attraction, alarm message transmission, and so on [70,71]. 2,5-DMP emitted by *Pseudomonas putida* BP25 had the ability to enhance anthracnose resistance and extend the shelf life of mangos [72].

### 2.3. Trimethylpyrazine

**Figure 2:**
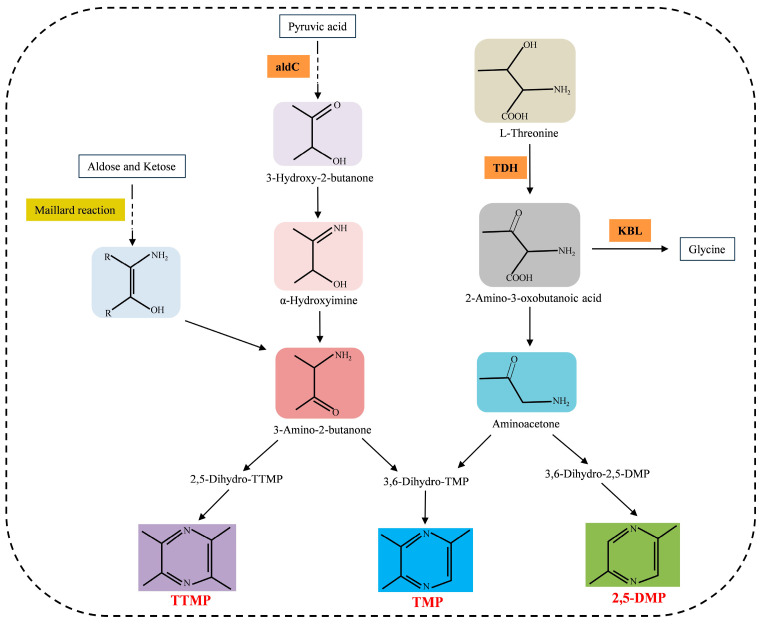
The possible synthesis pathways of TTMP, TMP, and 2,5-DMP.

As another important alkylpyrazine, 2,3,5-trimethylpyrazine (TMP) consists of one pyrazine ring with three methyl groups (Figure 2) [73]. TMP is a kind of fancy spice which has gradually been developed in recent years; it possesses a strong aroma of roasted peanuts or potatoes. TMP exists in cocoa products, baked products, dairy products, coffee, peanuts, popcorn, and other foods, and it is a kind of important raw material for flavorings and tobaccos. TMP can be directly used in the food industry, including in products such as bread, pudding, chewing gum, soft drinks, milk, meat, and so on [74]. TMP has been isolated and identified in several types of the Chinese liquor *Baijiu*, Scotch-blend whiskey, and a Jamaican rum [20,73,75].

#### The Potential Functions of TMP

It has been reported that TMP is a kind of sex pheromone in the rectum of male fruit flies which contributed to attracting mature female flies [76,77]. Zhang et al. [78] showed that TMP, 2,5-DMP, and 2,3-DMP had the function of stabilizing the structure of 3,5-dichlorosalicylic acid (DCA) and pyrazine derivatives co-crystals, through changing the bonding mode and intermolecular force of the co-crystals. TMP as a by-product of succinic acid production was the most abundant compound in a waste culture supernatant, which became a sustainable resource in industrial waste utilization from succinic acid production [79]. In summary, reports on the synthesis pathway and potential function of TMP are still scarce.

## 3. Synthesis Pathways of Naturally Occurring Pyrazines

Naturally occurring pyrazines are mainly formed from the Maillard reaction or fermentation processes. The synthesis pathways of some pyrazines have been reported. Here, we summarized the possible synthesis pathways of TTMP, TMP, and 2,5-DMP (Figure 2) [2,80,81].

### 3.1. The Synthesis of TTMP

Currently, three main methods can be used for TTMP production. Firstly, TTMP can be directly extracted from medicinal plants, e.g., *L. wallichii* [29]. Chemical synthesis is also a technology for TTMP production, but it has become an unwilling choice on account of its environmental pollution, lacking relevant natural resources and having high costs during the production process of TTMP [82]. Thirdly, a route based on biosynthesis is an increasingly popular method for industrialized TTMP production because of its high production efficiency and environmentally friendly production process [34,83]. In the biosynthetic pathway of TTMP, acetoin (3-hydroxy-2-butanone) and ammonia are precursors of TTMP synthesis. Specifically, acetoin reacts with ammonia to form α-hydroxyimine; after that, α-hydroxyimine spontaneously converts to 2-amino-3-butanone and forms TTMP [31,33].

Due to the preference of natural products in applications of food and medicines, more attention has been paid to bio-based TTMP synthesis. Previous research has found out that several microorganism species (including *Corynebacterium glutamicum*, *Bacillus licheniformins*, *B. subtilis*) can metabolically generate acetoin and TTMP [33,83]. Nevertheless, rate-limiting steps exist in TTMP biosynthesis pathways, resulting in a low synthesis efficiency and low yield [84]. Therefore, more attempts have been made to solve these problems through breeding high-producing strains, optimizing growth conditions, screening efficient mediums, and so on [31,33,85] (Table 3).

Xu et al. [33] used a combinational optimization method to efficiently produce TTMP. Specifically, they generated a recombinant strain (*Escherichia coli* BL15) containing genes (*transcriptional regulator* (*alsR*), *acetolactate synthase* (*alsS*), *acetolactate decarboxylase* (*alsD*)) and optimized the expression of the *NADH oxidase* (*nox*) gene [33]. Afterwards, they regulated the medium formulation and aeration rate, combined with the adjustment of pH values for fermentation [33]. Studies on Chinese sesame-flavor liquor showed that TTMP was mainly generated during distillation, solid-state fermentation, and stack fermentation, and a higher temperature during fermentation was conducive to the efficient production of TTMP [31]. Zhang et al. [86] isolated and identified a TTMP-producing strain, *B. amyloliquefaciens* XJB-104, which was used to generate TTMP through solid-state fermentation with distillers’ grains as a medium. The results showed that the yield of TTMP reached 1.28 × 10^3^ mg/kg, which was 6.3 times higher than the control [86]. The thermophilic bacterium *Laceyella sacchari* FBKL4.010 isolated from Moutai-flavor *Daqu* was prepared for whole genome sequencing and analyzed by Li et al. [87]. They found that a set of genes was involved in TTMP synthesis, and they were remarkably reordered in the *L. sacchari* FBKL4.010 strain [87]. Meng et al. [85] reported that over-expression of the *α-acetolactate decarboxylase* (*aldC*) gene in *B. licheniformis* BL1 had a higher TTMP yield compared to the initial *B. licheniformis* BL1 strain. In addition, the application of exogenous acetaldehyde can enhance the output of TTMP and acetoin during the fermentation process in an initial *B. licheniformis* BL1 strain [85]. Research on *Saccharomyces cerevisiae* showed that disrupting the *2,3-butanediol dehydrogenase 1* (*BDH1*) gene and overexpressing the *BDH2* gene can significantly elevate TTMP production during *Baijiu* brewing [88]. Furthermore, by constructing diploid strains AY-SB1 and AY-SD1B2, the yield of TTMP had around a 2-3-fold increase compared to the initial strain [88]. An industrially relevant strain *Corynebacterium glutamicum* ATCC 13,032 engineered with heterologous genes was used to analyze TTMP biosynthesis [89]. The results suggested that the accumulation of TTMP and acetoin in the constructed strain was caused by the feedback from a heterologous gene of the host strain [89]. In addition, ionic liquids as a pretreatment reagent can stimulate the production of TTMP in constructed strains [89]. By impairing or blocking the metabolic pathways of by-products in the biosynthesis of TTMP, Li et al. [34] constructed a novel green route to improve the yield of TTMP in a recombinant *E. coli* strain.

### 3.2. The Synthesis of 2,5-DMP

Currently, chemical synthesis and bio-based synthesis are common methods for 2,5-DMP production. However, due to the fact that shortcomings of chemical synthesis are difficult to overcome, and the fact that the by-products can even be toxic, biosynthesis approaches have attracted more attention for industrialized 2,5-DMP production [60,90].

Scientists have begun to work on raising the yield of 2,5-DMP by mainly focusing on two aspects: genetic modification and fermentation condition optimization (Table 4). The synthesis mechanisms of 2,5-DMP were studied by Zhang et al. [73]. A couple of experiments were performed and the results suggested that L-threonine was a starting point to form 2,5-DMP [73]. This 2,5-DMP biosynthesis pathway includes one enzymatic reaction catalyzed by L-threonine-3-dehydrogenase (TDH) and three non-enzymatic reactions [73]. Inactivation of 2-Amino-3-ketobutyrate coenzyme A (CoA) ligase (KBL) improved the yield of 2,5-DMP, since KBL competitively inhibited the synthesis of 2,5-DMP by catalyzing the cleavage of L-2-amino-acetoacetate, an intermediate in the 2,5-DMP biosynthesis pathway [73]. Xu et al. [91] rewrote the pathway of *de novo* 2,5-DMP biosynthesis in a high L-threonine strain, resulting in high 2,5-DMP production from glucose after submerged fermentation. A recombinant *E. coli* strain was constructed to obtain high efficiency when converting L-threonine to 2,5-DMP [92]. Concretely, the *TDH* gene from *E. coli* and the *NADH oxidase* (*Nox*) gene from *Lactococcus cremoris* were modified and co-expressed in *E. coli* cells, which reduced the production of by-products by disrupting original metabolic pathways and increased the conversion ratio of L-threonine, resulting in a high yield of 2,5-DMP [92]. More recently, the *TDH* gene from *E. coli* and the *Nox* gene from *Enterococcus faecium* were overexpressed in *E. coli* using the pACYCDuet-1 expression system [93]. The recombinant *E. coli* cells can convert L-threonine to produce 2,5-DMP with a yield of 2009.31 mg/L [93]. Similarly, the engineering strain modified by using the *EcTDH* gene, the *L-threonine transporter protein* (*EcSstT*) gene from *E. coli* BL21, the *EhNox* gene from *E. hirae*, and the *aminoacetone oxidase* (*ScAAO*) gene from *Streptococcus cristatus* produced 2897.30 mg/L 2,5-DMP after optimizing the reaction conditions [94]. Zeng et al. [95] constructed an engineering strain of *E. coli*, which can produce 2,5-DMP without the need for inducers or antibiotics. With glucose as the carbon source, the yield of DMP can reach 3.1 g/L after fermentation [95]. In another study, the *AAO* gene from *S. oligofermentans* was introduced and overexpressed in *E. coli*, together with overexpression of the *EcTDH* gene [60]. Simultaneously, the *2-amino-3-ketobutyrate CoA ligase* (*KBL*) gene encoding a key enzyme in competitive branching metabolic pathways of 2,5-DMP biosynthesis was knocked out in *E. coli* [60]. As a result, the reconstructed *E. coli* strain obtained a high carbon recovery rate (30.18%) and high 2,5-DMP production through enhancing enzymatic and non-enzymatic reactions and reducing bypass waste [60]. It has been reported that 2,5-DMP contributed to the formation of a roasted peanutty flavor in Oolong tea [56]. Furthermore, efforts have been made to demonstrate that L-theanine played a role in the generation of 2,5-DMP during the manufacturing process in Oolong tea [56]. Research on baked food products showed that exogenously adding two whey protein hydrolysates increased the level of 2,5-DMP production, which was formed from hydrolysis of proteinase or trypsin in *Aspergillus melleus* [11]. A stable isotope dilution assay was performed to verify the function of whey protein hydrolysates in the formation of 2,5-DMP in bread and cookies, and the results showed that the small quantities of whey protein hydrolysates evidently promoted the formation of 2,5-DMP on account of the peptides contained, whereas high amounts caused negative effects in terms of the appearance and aroma [11]. The latest research on 2,5-DMP biosynthesis has been elaborated above, although more studies on 2,5-DMP have been focused on chemistry. The relevant content on chemistry is not covered in this review.

### 3.3. The Synthesis of TMP

Lu et al. [96] reported a degradation profile of oligopeptides in a Maillard reaction system, which showed that a reaction with glucose and glycine/diglycine/triglycine conducted in mediums with different water contents can generate TMP and 2,5-DMP. Furthermore, to a certain degree, the water content decreased, leading to enhanced production of TMP [96]. TMP can be formed by Maillard reactions, with glucose, methylglyoxal, or glyoxal providing carbon and dipeptides with lysine at the N terminus providing the nitrogen [97,98]. Wang et al. [99] established several Maillard reaction models by using glucose and different peptides. TMP, 2,5-DMP, and 2,6-DMP are the most abundant pyrazine compounds in models using dipeptide (such as Arg-Lys, His-Lys, Lys-His, and Lys-Arg). Their results proved that the peptide structure played a key role in the formation of pyrazine during the Maillard reaction [99]. Carbon module labeling technology and model response validation experiments performed by Jiang et al. [100] showed that the methylglyoxal and glyoxal conversion reaction was an important way to transform formaldehyde carbon into the methyl group carbon of TMP. Maillard reactions occurred during the roasting process of large-leaf yellow tea and the results of one study showed that lysine together with D-glucose contributed significantly to the formation of TMP [101].

In recent decades, TMP derived from microbial fermentation has become more and more popular. However, the yield of TMP synthesis by microorganisms is relatively low [80,102]. It has been reported that bacteria such as *Bacillus licheniformis*, *B. subtilis*, *B. amyloliquefaciens*, *B. sonorensis*, and *Lactococcus lactis* can produce TMP [14,103]. Liu et al. [81] isolated a *Bacillus amyloliquefaciens* strain from *Daqu*, which can produce TMP after solid-state fermentation. Under optimized fermentation conditions, the yield of TMP reached 0.446 ± 0.052 mg/g, which was about five times higher than the yield obtained before optimization [81]. In *Bacillus subtilis* 168, TDH can catalyze the reaction of D-glucose and L-threonine to produce TMP [73]. In addition, Zhang et al. [73] found that exogenous L-threonine and ammonium salt can also produce TMP in the absence of D-glucose in whole-cell catalytic experiments. In vitro experiments showed that providing exogenous threonine and glucose as substrates can significantly enhance the production of 2,3,5-TMP in the *Bacillus* species isolated from the rectum of the male oriental fruit fly [104].

## 4. Concluding Remarks and Future Perspectives

Pyrazines are important flavor substances in food. Most pyrazines have a relatively low threshold, but they indeed have a great impact on food flavor and quality. The understanding of the formation of pyrazines in fermented food needs to be strengthened and deepened to obtain high-quality functional foods and beverages.

**Figure 3:**
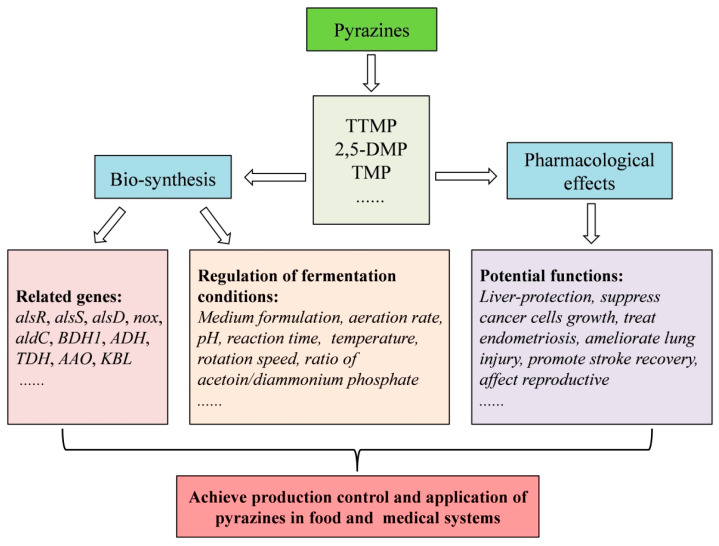
Summary of biosynthesis and pharmacological effects of three essential pyrazines.

This review considers the three essential pyrazines, TTMP, 2,5-DMP, and TMP, focusing on their characteristics, possible synthetic pathways, and potential functions, in particular biosynthesis and medical effects (Figure 3). Recent progress in the research of pyrazines, especially regarding synthesis and applications, has also been discussed, providing a reference for further study and industrialization of pyrazines. Currently, the main challenge is how to develop controllable and continuable strategies to promote industrialized production of pyrazines and simultaneously minimize potential harmful by-products in the food industry.

Some recent methods for elevating the production of TTMP, 2,5-DMP, and TMP are summarized in this review, separately. Most of the research only focused on one factor and studied its effect on the yield of corresponding pyrazines. Actually, the synthesis of pyrazines is often affected by many factors simultaneously. Therefore, to increase the production of pyrazines to a greater extent, different variables should be considered using statistical designs in future studies. In addition, most studies on the medical efficacy of TTMP are performed on experimental animals under laboratory conditions. So, there is still a long way to go before we can realize its medical value.

## Figures and Tables

**Table 1:** Examples of potential medical effects of TTMP.

Roles of TTMP	Sources of TTMP	Mechanisms	Experimental Species	References
Alleviate the symptoms of Alzheimer’s disease	ND ^1^	Reduce beta-amyloid levels and tau phosphorylation; change the expression of oxidative phosphorylation complex proteins in the hippocampus	Alzheimer’s disease transgenic mice	[36]
Attenuate pulmonary hypertension	Purchased from Sigma-Aldrich (St. Louis, MO, USA)	Antioxidant properties	Monocrotaline-induced pulmonary hypertension rats; pulmonary cells from humans	[37]
Alleviate behavioral and psychological symptoms of dementia	Purchased from Sigma-Aldrich (St. Louis, MO, USA)	Alleviate dendritic and spine deficits; up-regulate the expression of synapse-related proteins in the hippocampus; activate signaling pathways to promote synaptic remodeling	Male Sprague Dawley rats	[38]
Liver-protective activity	Produced by *Bacillus amyloliquefaciens* XJB-104 with distillers’ grains	Decrease serum levels of biochemical indicators of liver injury and suppress inflammatory cytokines; alleviating the decrease in SOD ^2^, CAT ^3^, GSH ^4^, and MDA ^5^	Kunming male mice	[39]
Treat endometriosis	Purchased from Sigma-Aldrich (St. Louis, MO, USA)	Decrease lesional fibrosis and lesion weight; improve hyperalgesia	Virgin female Balb/C mice; endometriotic tissue from 5 premenopausal cycling women	[40]
Suppress ovarian tumor cells’ proliferation	Purchased from Energy Chemical Co., Ltd. (Shanghai, China)	Suppress angiogenesis; promote apoptosis of tumor cells; augment the antitumor effects of paclitaxel	Female BALB/c nude mice	[41]
Suppress cancer cells’ growth	Purchased from Saeng Chemical Co., Ltd. (Shanghai, China)	Higher cytotoxicity, apoptosis rate, and cell-cycle arrest (conjugated with paclitaxel); promote cellular uptake and intracellular drug release	Female nu/nu nude mice	[42]
Treat spinal cord injury	Purchased from Sigma-Aldrich (St. Louis, MO, USA)	Anti-inflammatory, antioxidant, and neuroprotective activity	Male specific pathogen-free Sprague Dawley rats	[43]
Promote functional outcome after ischemic stroke	Purchased from Aladdin Chemistry Co. (Shanghai, China)	Ameliorate synaptic interface and postsynaptic density; partially participate in regulation of neuroplasticity	Male Sprague Dawley rats	[44]
Promote stroke recovery	Purchased from Harbin Medisan Pharmaceutical Co., Ltd. (Lot No. 090923A, Harbin, Heilongjiang, China)	Induce the restoration of neurovascular unit and transformation of A1/A2 reactive astrocytes	Male Sprague Dawley rats	[45]
Ameliorate acute lung injury	Obtained from the National Institute for the Control of Pharmaceutical and Biological Products (Beijing, China)	Regulate the Rac1/LIMK1 signaling pathway	C57/BL6 male mice	[46]

^1^: ND, no data; ^2^: SOD, superoxide dismutase; ^3^: CAT, catalase; ^4^: GSH, reduced glutathione; ^5^: MDA, malondialdehyde.

**Table 2:** Examples of potential medical effects of 2,5-DMP.

Effects of 2,5-DMP	Sources of 2,5-DMP	Mechanisms	Experimental Species	References
Affect reproductive and accessory reproductive organs	ND ^1^	Reduce uterus weight	Female rats	[61]
Affect reproductive and accessory reproductive organs	ND	Decrease the weight of prostate, seminal vesicles, and testosterone levels in plasma	Male rats	[62]
Affect plasma testosterone and polyamines and acid phosphatase levels	ND	Decrease plasma testosterone and levels of polyamines and acid phosphatase in the prostate	Juvenile male rats	[63]
Affect uterine contraction	ND	Inhibit the late phase of pregnant uterine movements	Female rats	[65]
Induce sperm-head abnormalities	Purchased from Sigma-Aldrich (St. Louis, MO, USA); released by female CBA mice	Induce genetic damage during meiotic divisions	Highly inbred male CBA mice	[66]
Affect the concentration of plasma non-esterified fatty acid	ND	Suppress the non-esterified fatty acid rebound induced by nicotinic acid	Male Wistar rats	[67]
Affect the concentration of blood lipids	ND	Decrease blood lipid concentrations	Male Wistar rats	[68]

^1^: ND, no data.

**Table 3:** List of studies on TTMP biosynthesis.

Genes	Experimental Species	Measures	TTMP Yield	References
*Transcriptional regulator (alsR)*, *acetolactate synthase (alsS)*, *acetolactate decarboxylase (alsD)*, *NADH oxidase (nox)*	*Escherichia coli* BL15	Regulate medium formulation and aeration rate; adjust pH values and ammonium salt for fermentation	16.10 g/L	[33]
*Diacetyl reductase* gene, *fumarate reductase* gene, *lactate dehydrogenase* gene, etc.	*E. coli*	Impair or block genes of by-products metabolic pathways; optimize molar ratio of acetoin/diammonium phosphate, reaction time, temperature and rotation speed of the microreactor	56.72 g/L	[34]
*α-acetolactate decarboxylase (aldC)*	*Bacillus licheniformis* BL1	Overexpress *aldC* gene; supply exogenous acetaldehyde in fermentation medium	43.75 g/L	[85]
ND ^1^	*B. amyloliquefaciens* XJB-104	Adjust the amount of distillers’ grains during solid-state fermentation; adjust pH; improve Fuqu medium	1.28 g/kg	[86]
*Amino acid dehydrogenase (ADH)*, *transaminase gene*	*Laceyella sacchari* FBKL4.010	Analyze TTMP metabolism related gene	ND	[87]
*2,3-butanediol dehydrogenase 1* (*BDH1*) and *BDH2*	*Saccharomyces cerevisiae*	Disrupt *BDH1* and overexpress *BDH2*	9.47 mg/L	[88]
*mk* and *hmgR* homologs	*Corynebacterium glutamicum* ATCC 13032	Express *mk* homologs (from *Staphylococcus aureus* and *Corynebacterium kroppenstedtii*) and *hmgR* from *S. aureus*; Feed with ionic liquids	5.00 g/L	[89]

^1^: ND, no data.

**Table 4:** List of studies on 2,5-DMP biosynthesis.

Genes	Experimental Species	Measures	2,5-DMP Yield	References
ND ^1^	*Aspergillus melleus*	Add two whey protein hydrolysates	ND	[11]
ND	Oolong Tea *(Camellia sinensis)*	Add additional L-theanine	ND	[56]
*L-threonine-3-dehydrogenase (TDH)*, *aminoacetone oxidase (AAO)*, *2-Amino-3-ketobutyrate coenzyme A (CoA) ligase (KBL)*	*Escherichia coli*	Overexpress *TDH* and *AAO*; knock out *KBL* gene	1682.00 mg/L	[60]
*TDH*, *KBL*	*Bacillus subtilis* 168	Heterologously express *TDH*; inactivate *KBL*; supply with L-threonine	2.82 mM/L	[73]
*TDH; AAO; NADH oxidase (Nox); L-threonine transporter protein (SstT)*	*E. coli*	Rewrite the *de novo* 2,5-DMP biosynthesis pathway; supply with L-threonine; balance the intracellular NADH/NAD+ ratio; control the transmembrane transport of L-threonine	1.43 g/L	[91]
*TDH*, *Nox*	*E. coli* BL21(DE3)	Overexpress *TDH* gene; optimize the expression mode of *Nox*; disrupt shunt metabolic pathway to reduce by-products	1095.70 mg/L	[92]
*TDH*, *Nox*	*E. coli* BL21(DE3)	Overexpress *EcTDH* and *EfNox*; add additional L-threonine	2009.31 mg/L	[93]
*TDH*, *SstT*, *NoX*, *AAO*	*E. coli* BL21(DE3)	Overexpress *EcTDH*, *EcSstT*, *EhNox*, and *ScAAO*; optimize reaction conditions	2897.30 mg/L	[94]
*TDH*	*E. coli*	Enhance *TDH* gene expression; modify the L-threonine transport system	3.1 g/L	[95]

^1^: ND, no data.
